# LiCl induces apoptosis via CHOP/NOXA/Mcl-1 axis in human choroidal melanoma cells

**DOI:** 10.1186/s12935-021-01778-2

**Published:** 2021-02-08

**Authors:** Qiuqiu Zhang, Qianwei Zhang, Huiyuan Li, Xiaofei Zhao, Han Zhang

**Affiliations:** 1 Department of Ophthalmology, The Second Hospital, Cheeloo College of Medicine, Shandong University, 247 Beiyuan Street, Jinan, Shandong Province 250033 China; 2 Department of Ophthalmology, Zaozhuang Municipal Hospital, Zaozhuang, Shandong China 277100; 3 Department of Ophthalmology, Jinan Central Hospital, Cheeloo College of Medicine, Shandong University, Jinan, Shandong 250013 China

**Keywords:** Human choroidal melanoma, LiCl, Apoptosis, Endoplasmic reticulum stress, NOXA/Mcl-1

## Abstract

**Background:**

Choroidal melanoma is the most common primary intraocular malignancy that occurs in adults. Lithium Chloride Promotes Apoptosis in Human Leukemia NB4 Cells by Inhibiting Glycogen Synthase Kinase-3 Beta. In this study, we aimed to understand whether LiCl exerts anticancer effects on choroidal melanoma cells and elucidate the underlying molecular mechanisms.

**Methods:**

Human choroidal melanoma cells were treated with LiCl, and cell survival was assessed with MTT assays. Cell reproductive viability was measured by plate colony formation assays. Cell apoptosis was evaluated using flow cytometry, and proteins were detected using western blotting. A human choroidal melanoma xenograft model was established to demonstrate the effect of LiCl on human choroidal melanoma in vivo.

**Results:**

We found that LiCl inhibited cell survival and clonogenic potential and induced apoptosis in human choroidal melanoma cells. LiCl also reduced the proliferation of choroidal melanoma cells in vivo. Moreover, the upregulation of NOXA and downregulation of Mcl-1 were responsible for LiCl-induced apoptosis. Mcl-1 overexpression obviously impaired LiCl-induced apoptosis and cleavage of caspase8, caspase9, caspase3 and PARP. Moreover, the protein expression of endoplasmic reticulum stress markers, including IRE1α, Bip, p-eIF2α, ATF4 and CHOP, were upregulated following treatment with LiCl. When CHOP expression was knocked down and cells were treated with LiCl, the protein level of NOXA was partially increased, and Mcl-1 expression was increased, while the cleavage of caspase8, caspase9, caspase3 and PARP that was induced by the LiCl was reduced compared with the vehicle treated group. Prolonged ER stress results in the activation of the apoptotic pathway.

**Conclusions:**

In summary, LiCl induced an endoplasmic reticulum stress response while activating intrinsic apoptosis. Furthermore, the CHOP/NOXA/Mcl-1 axis contributed to LiCl-induced apoptosis both in vitro and in vivo. The present study provides important mechanistic insight into potential cancer treatments involving LiCl and enhances the understanding of human choroidal melanoma.

## Background

Choroidal melanoma is the most common primary intraocular malignancy that occurs in adults. The incidence of malignant melanoma is increasing worldwide [1]. Despite successful eradication of the tumour, up to 50% of choroidal melanoma patients develop metastatic disease (typically to the liver) [2]. Due to its latency and metastatic potential, the mortality of choroidal melanoma is approximately 50% [3]. Once choroidal melanoma metastasizes, the median survival time after detection of the first metastasis is 8 months [4]. Poor prognosis is related to various molecular factors, but the mechanisms of choroidal melanoma development remain to be elucidated.

Enucleation, phototherapy, and various forms of radiotherapy are currently common treatments for choroidal melanoma [5]. However, present treatments offer only temporary relief and have several shortcomings, such as neovascular glaucoma, retinal detachment and tumour recurrence [6]. And the effect of enucleation of the Choroidal melanoma is controversy. Some researchers believed that microscopic metastasis was already present before enucleation or even at time of diagnosis of choroidal melanoma [7]. Without effective treatment, patients are threatened with pain, visual handicap, and facial disfigurement. Therefore, exploring new treatment strategies and improved prognosis of choroidal melanoma patients is a matter of great urgency [8].

Lithium compounds have been used in the clinic to treat the acute symptoms of bipolar diseases for several decades [9]. Lithium chloride (LiCl), an inhibitor of GSK3β that promotes GSK3β phosphorylation and inactivation, has been safely used in the clinic for the treatment of psychiatric disorders for many years [10, 11]. It has also been reported that LiCl can induce apoptosis in pancreatic ductal malignant gland cells and G2/M cell cycle arrest in liver cancer and non-small cell lung cancer [12]. In addition, lithium also plays a role as an adjuvant for radiation and chemotherapy, while increasing evidence has confirmed the value of combination treatment with lithium for cancers [13]. As an activator of the Wnt/β-catenin signalling, LiCl can elevate the accumulation of β-catenin and trigger the activation of Wnt/β-catenin pathway. Moreover, transcriptionally active β-catenin is associated with less invasive disease and more favorable prognosis for melanoma patients, compare to other cancers, in which nuclear β-catenin is a driving force of both initiation and progression [14]. However, whether LiCl exhibits an anticancer effect on choroidal melanoma is still unknown. And exploring the underlying molecular mechanism is important for developing novel effective therapies for choroidal melanoma.

In this study, we investigated whether LiCl exerts apoptotic effects on choroidal melanoma cells. We also explored the role of Mcl-1and NOXA as regulators of this effect. Our findings suggested that LiCl is a promising candidate therapy for the treatment of choroidal melanoma and enriches our understanding of the underlying molecular mechanisms for LiCl-induced choroidal melanoma cell apoptosis.

## Materials and methods

### Antibodies and reagents

LiCl was purchased from Sigma-Aldrich (St. Louis, MO, USA). Antibodies against caspase8 (cat no. 9746), caspase9 (cat no. 9502), Bip (cat no. 3183), poly (ADP-ribose) polymerase (PARP; cat no. 9542), phospho-eIF2α (Ser51) (D9G8) (cat no. 3398S), eIF2α (cat no. 9722S), IRE1α (cat no. 3294), ATF-4 (D4B8) (cat no. 11815S) were purchased from Cell Signaling Technology (Danvers, MA, USA). Antibodies targeting caspase3 (cat no. NB100-56708) were purchased from Imgenex (Novus Biologicals, LLC, Littleton, CO, USA). The NOXA antibody (cat no. OP180) was obtained from Calbiochem (Merck KGaA, Darmstadt, Germany). Antibodies against CHOP (cat no. sc-7351) and Mcl-1 (cat no. sc-12756) were purchased from Santa Cruz Biotechnology, Inc. (Dallas, TX, USA). The GSK3β antibody was obtained from Abcam (cat no. ab32391; Abcam; UK).

### Cell lines and cell culture

The human choroidal melanoma lines OCM1 and M619 were obtained from the China Centre for Type Culture Collection (Wuhan, China) and were grown in monolayer cultures at 37 °C in a humidified atmosphere consisting of 5% CO_2_ and 95% air. OCM1 cells were cultured in Dulbecco’s modified Eagle’s medium containing 5% foetal bovine serum, and M619 cells were cultured in RPMI-1640 medium containing 5% foetal bovine serum (both Gibco; Thermo Fisher Scientific Inc., Waltham, MA, USA).

### Cell viability assay

Cells were seeded in 96-well plates at a density of 5.0 × 10^3^ cells/well and were then treated with the indicated concentrations of LiCl on the second day. The cells were cultured with chemotherapeutics for 24, 36 or 48 h then subjected to the MTT assay. Each sample was incubated with 20 µl of (5 mg/ml) MTT (Sigma–Aldrich; Merck KGaA) at 37 °C for 4 h. Then, the solution was discarded, and 100 μl of dimethyl sulfoxide was added. The absorbance at 495 nm due to formazan was measured by an ELISA Multiskan reader (Thermo Fisher Scientific, Inc.).

### Colony formation assay

The cells were seeded into 6-well plates at a density of 1 × 10^4^ cells per well. After the cells were incubated overnight, the cells were treated with 0, 2.5, 5, or 10 mM LiCl and incubated for approximately 2 weeks. During this period, the indicated concentrations of LiCl were added to the wells every 72 h. When the cell colonies were visible to the naked eye, the cells were subjected to the colony formation assay. The culture solution was discarded, and the cells were washed twice with phosphate-buffered saline (PBS) and fixed with 4% paraformaldehyde for 20 min. After that, the cells were washed with PBS 3 times, stained with 1% crystal violet for 20 min, washed out slowly with water, and dried in the air. The number of cell colonies (> 50 cells) was counted under a microscope.

### Apoptosis assay

Apoptosis was evaluated according to a previously described protocol [15]. The Annexin V-FITC/propidium iodide (PI) apoptosis detection kit was purchased from BIO-BOX Biotech (Nanjing, China). The cells were treated with various concentrations of LiCl for 36 h, and then 2 × 10^6^ cells were collected, washed with prechilled PBS and resuspended in 500 μl of binding buffer. Then, each sample was incubated with 5 μl of Annexin V-FITC and 5 μl of PI for 15 min in the dark at room temperature. Then, the cells were analysed in a FACScan flow cytometer (Becton–Dickinson, San Jose, CA, USA). Data analysis was performed using FlowJo software (version 7.2.2; Tree Star, Inc. San Carlos, CA, USA).

### Western blotting analysis

Whole-cell protein lysates were prepared and analysed by western blotting according to a previously described protocol [15]. After being harvested and rinsed with prechilled PBS, the cells were lysed, and the extract was centrifuged at 12,000x*g* at 4 °C for 15 min. Whole–cell protein lysates (40 µg) were electrophoresed on 12% denaturing polyacrylamide slab gels and transferred to Hybond-enhanced chemiluminescence (ECL) membranes through electroblotting. The membranes were blocked with 5% nonfat milk for 1 h at room temperature and then probed with specific primary antibodies and subsequently with secondary antibodies. Antibody binding was detected using an ECL system (EMD Millipore, Billerica, MA, USA) according to the manufacturer’s protocol. The protein expression levels were quantified using ImageJ software (version 1.6.0_24; National Institute of Health, Bethesda, MD, USA).

### Plasmid transient transfection

The pcDNA3.1-Mcl-1 plasmid was obtained from Addgene (Cambridge, MA, USA). OCM1 and M619 cells were seeded in 6-well plates and transfected with pcDNA3.1 and pcDNA3.1-Mcl-1 plasmids using X-treme GENE HP DNA Transfection Reagent (Roche Molecular Biochemicals, Mannheim, Germany) according to the manufacturer’s protocol. Then, the cells were treated with the indicated concentration of LiCl for 24 h and subjected to western blotting and apoptosis analysis.

### Transfection with siRNA

Previously described siRNAs targeting sequences of CHOP and GSK3β were synthesized [15, 16]. Transfection with siRNA was conducted using jetPRIME Transfection Reagent (Polyplus Transfection SA, Illkirch, France) following the manufacturer’s protocol. Choroidal melanoma cells were seeded in 6-well plates and transfected with control or target siRNA on the second day. Two days later, the cells were treated with various concentration of LiCl for another 24 h. Then the cells were harvested for western blotting analysis.

### In vivo tumorigenesis analysis

Five-week-old BALB/c nude male mice were obtained from Beijing Vital River Laboratory Animal Technology (Co., Ltd./Charles River Laboratories, Beijing, China). The BALB/c nude male mice were randomly divided into a normal saline group and a LiCl group with 5 mice per group. M619 cells were subcutaneously injected into the right flank region of each mouse (3 × 10^6^ cells in 100 μl of PBS). The tumour size was recorded every 3 days beginning on the day the tumours were first visible. Tumour volume was calculated using the following equation: Volume = (width^2^ × lenghth)/2. The LiCl group was treated with LiCl (141.3 mg/kg; i.p., daily) for 2 weeks, while the normal saline group received an equal volume of normal saline. Finally, the mice were sacrificed on the 15th day, and the tumour tissues were collected for western blotting and immunohistochemical analysis. The study was approved by the Shandong University Second Hospital Ethics Committee.

### Immunohistochemical analysis

Immunohistochemical (IHC) analysis was performed according to a previously described protocol [17]. The tumour tissues were fixed in 10% formalin. Following proper dehydration, the tumours were embedded in paraffin and then cut into 5-μm-thick sections. After deparaffinization and rehydration, the sections were submerged in sodium citrate antigen retrieval solution (pH 6.0) and microwaved for 8–15 min for antigen retrieval. Endogenous peroxidase was deactivated by H_2_O_2_. Then, the slides were blocked using 10% goat serum and incubated in the corresponding primary antibodies overnight at 4 °C. After being washed, the sections were incubated with HRP-conjugated secondary antibody for 50 min at room temperature, followed by incubation with 3,3-diaminobenzidine (DAB) solution and counterstaining with haematoxylin. The anti-Ki67 rabbit mAb (cat no. GB 13030-2) was purchased from Wuhan Servicebio Technology Co., Ltd. (China).

### Statistical analysis

All experiments were repeated at least three times. All statistical analyses were performed using SPSS statistical software (version 20.0; IBM Corp., Armonk, NY, USA). The data are represented as the mean ± S.D. of at least three independent assays performed in duplicate or triplicate. An unpaired *t* test was used to compare differences between two groups, and one-way ANOVA was used to compare differences among more than two groups. A value of P < 0.05 was considered statistically significant.

## Results

### LiCl inhibits the survival and clonogenic potential of human choroidal melanoma cells

**Fig. 1:**
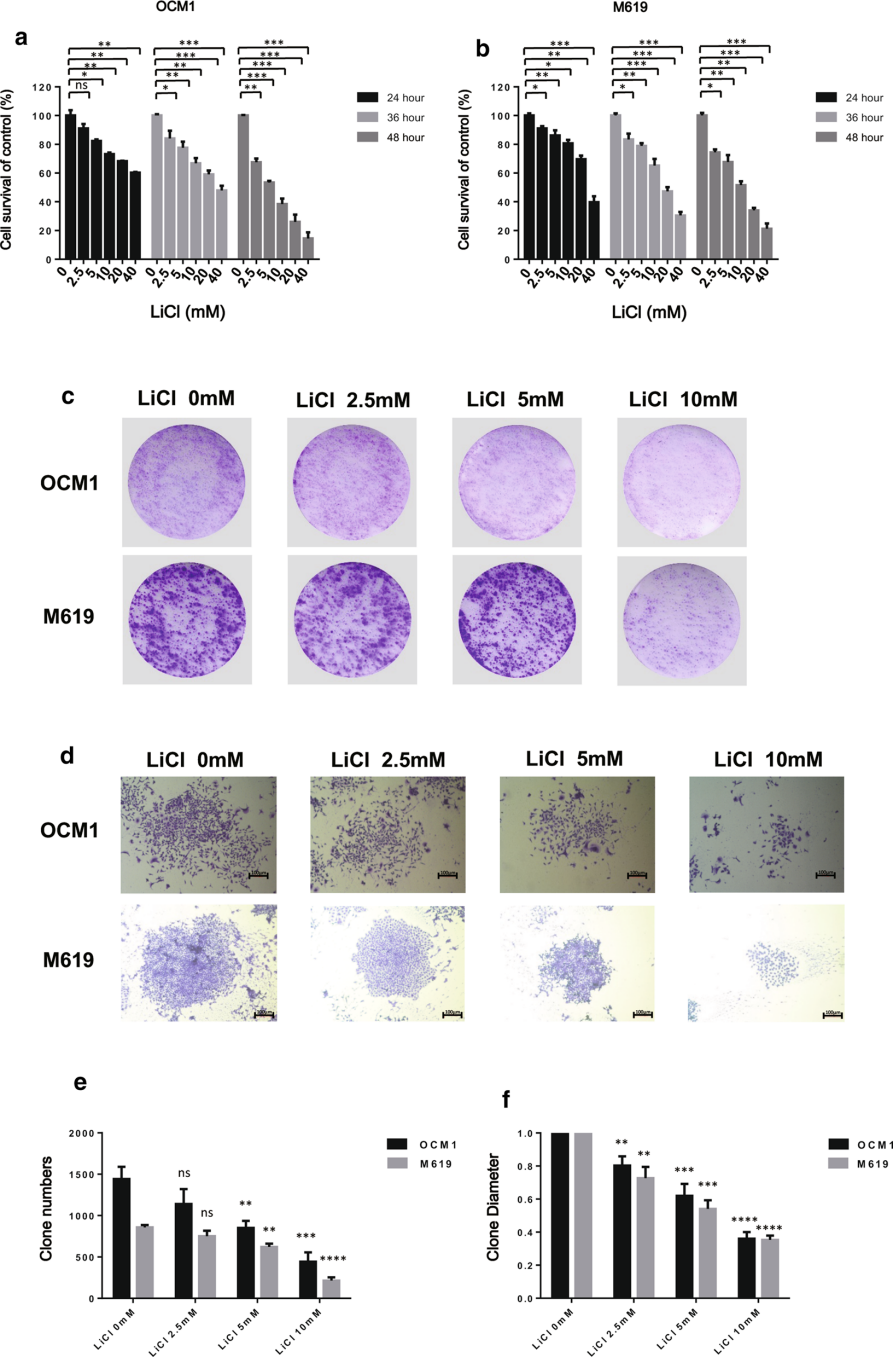
LiCl inhibits the survival and clonogenic potential of choroidal melanoma cells. **a** OCM1 and **b** M619 cells were seeded in 96-well plates, treated with 0, 2.5, 5, 10, 20 or 40 mM LiCl and incubated for 24 h, 36 h, 48 h. Cell survival was examined using the MTT assay. The survival rate at each drug concentration was compared with that of the normal saline group and analysed using SPSS software. All data are presented as the mean ± S.D. *P < 0.05,**P < 0.01,***P < 0.001. ns: not significant. **c** OCM1 and M619 cells were seeded in 6-well plates at a concentration of 1 × 10^4^ cells/well and cultured for approximately 2 weeks. Cells were treated with 0, 2.5, 5, or 10 mM LiCl, and various concentrations of LiCl were added to the wells every 72 h. When the cell colonies were visible to the naked eye, the culture was terminated, and the cells were stained with 1% crystal violet. **d** The size of the colonies. Magnification: × 100. Corresponding scale bars are depicted in the lower right corner of each image. Scale bars = 100 μm. **e** The number of clonies (> 50 cells) was counted under a microscope. **f** The diameter of a single colony was normalized to that in the vehicle-treated group. The experiments were repeated three times independently. All data are presented as the mean ± S.D. **P < 0.01, ***P < 0.001, ****P < 0.0001. *NS* not significant

To determine the cytotoxicity of LiCl in choroidal melanoma cells, OCM1 and M619 cells were treated with various concentrations of LiCl for different times. Effect of LiCl on cell viability was analysed by the MTT assay. As shown in Fig. 1a, b, LiCl inhibited the survival of human choroidal melanoma cells in a dose- and time-dependent manner. With increased concentrations and times, the cell viability decreased gradually. The IC50 values were calculated using GraphPad Prism 5.0 software. For OCM1 cells, the IC50 values of LiCl at 24 h, 36 h, 48 h were 93.8 mM, 40.25 mM, 6.25 mM respectively, while for M619 cells, the values were 39.02 mM, 22.36 mM 11.91 mM respectively. Cells were not decreased when the concentration of LiCl lower than the IC50 values especially LiCl was at low concentration. Other researchers found that LiCl could promote NB4 cells to apoptosis at the concentration of 20 mM while cells were not decreased with a low concentration of LiCl, which was consistent with our results [12]. Moreover, cell reproductive viability was measured by plate colony formation assays. As shown in Fig. 1c, d, choroidal melanoma cells were incubated with LiCl at various concentrations, the number and the size of colonies were not obviously decreased in the presence of 2.5 mM LiCl compared with that of the vehicle-treated cells. Statistical analysis showed that the number and the size of colonies of colonies were significantly decreased by 5 mM and 10 mM LiCl (Fig. 1e). These findings suggest that LiCl effectively suppressed the survival and clonogenic potential of human choroidal melanoma cells.

### LiCl triggers endoplasmic reticulum stress in choroidal melanoma cells

**Fig. 2:**
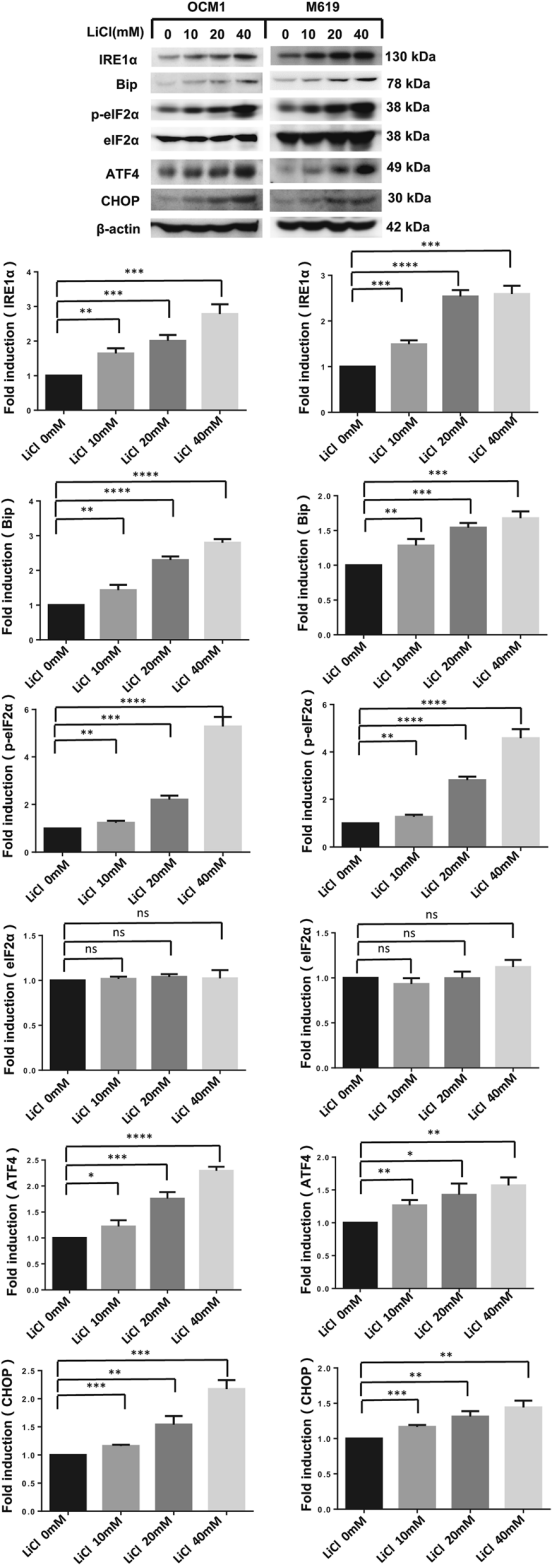
LiCl triggers endoplasmic reticulum stress in human choroidal melanoma cells. OCM1 and M619 cells were treated with 0, 10, 20, or 40 mM LiCl and incubated for 36 h. Following treatment, endoplasmic reticulum stress-related proteins were quantified by western blotting analysis. Protein expression was quantified using ImageJ software and analysed with GraphPad Prism 5.0 software. All data are presented as the mean ± S.D. *P < 0.05, **P < 0.01, ***P < 0.001, ****P < 0.0001. *NS* not significant

It has been demonstrated that the endoplasmic reticulum (ER) response is activated when cancers cells treated with chemotherapeutics [18]. We examined relevant proteins in the ER stress pathway to confirm whether LiCl triggers ER stress in choroidal melanoma cells. The western blotting results demonstrated that expression of the marker proteins IRE1α, Bip, p-eIF2α, ATF4 and CHOP were upregulated in a concentration-dependent manner after LiCl treatment (Fig. 2). These data indicate that LiCl triggers ER stress in choroidal melanoma cells.

### LiCl induces apoptosis in human choroidal melanoma cells

**Fig. 3:**
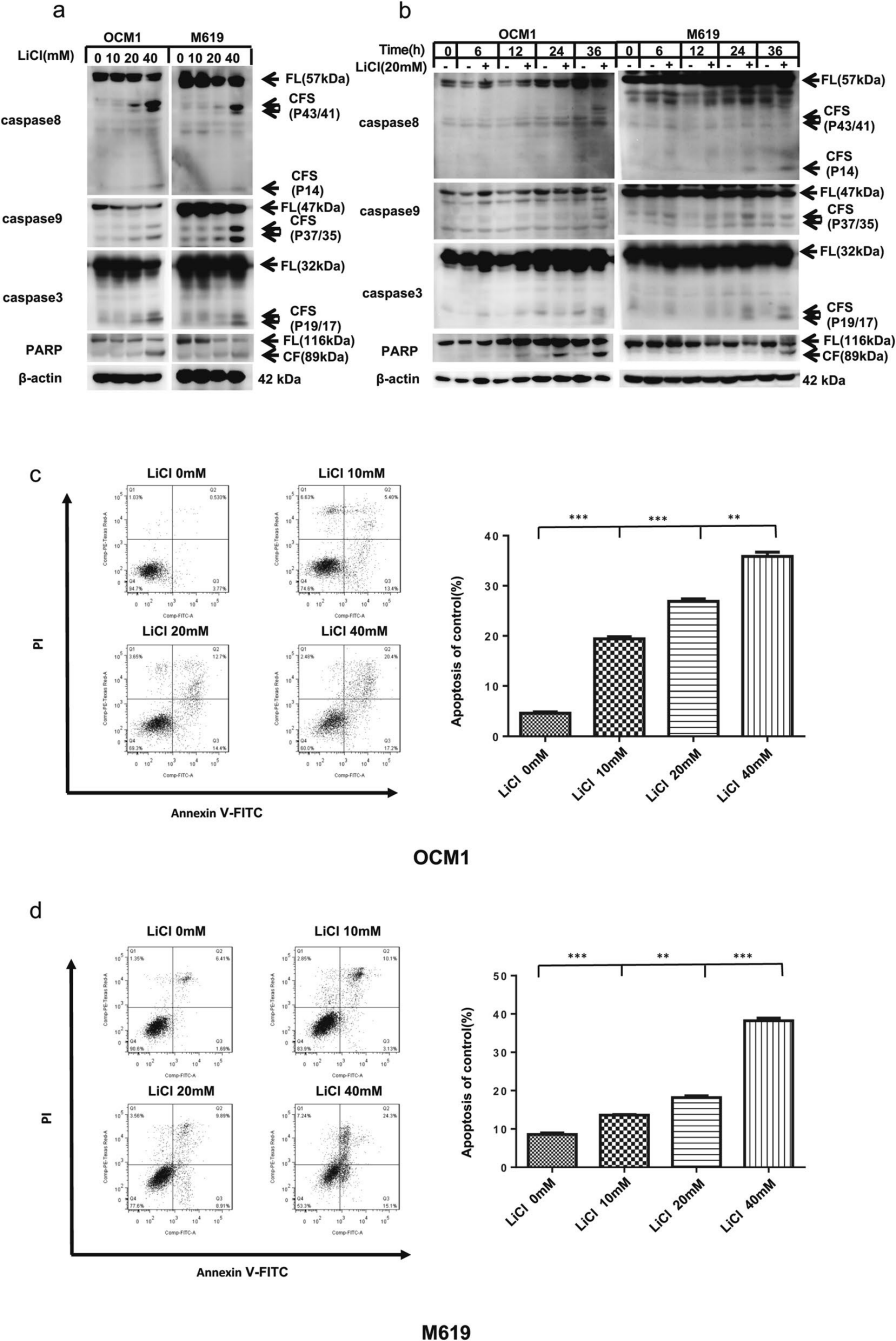
LiCl induces apoptosis through a caspase-dependent pathway in a concentration- and time-dependent manner. **a** To perform a dose-gradient assay, OCM1 and M619 cells were treated with 0, 2.5, 5, 10, 20 or 40 mM LiCl for 36 h and then harvested for western blotting analysis. **b** For the time-gradient assay, OCM1 and M619 cells were treated with 20 mM LiCl for 0, 6, 12, 24 and 36 h and then harvested for western blotting analysis. Apoptosis-related protein (caspase8 caspase9, caspase3 and PARP) expression was quantified using ImageJ software and analysed with GraphPad Prism 5.0 software. CF: cleaved form. CFs: plural of cleaved form. PARP: poly (ADP-ribose) polymerase. All data are presented as the mean ± S.D. **c d** Annexin V/PI staining was performed to evaluate the effect of LiCl on apoptosis. **c** OCM1 and **d** M619 cells were treated with 0, 10, 20 or 40 mM LiCl for 24 h and then harvested for apoptosis analysis. Data analysis was performed using FlowJo software and SPSS software. All data are presented as the mean ± S.D. *P < 0.05, **P < 0.001, ***P < 0.001. Q1: (Annexin V- FITC)-/PI + , necrotic cells. Q2: (Annexin V + FITC) +/PI + , late apoptotic cells. Q3: (Annexin V- FITC) +/PI-, early apoptotic cells. Q4: (Annexin V-FITC)-/PI-, normal vehicle-treated cells. PI, propidium iodide; FITC, fluorescein isothiocyanate

To determine whether apoptosis was involved in LiCl-induced inhibition of survival, we examined the expression of the cleavage of the apoptotic proteins. The western blotting results showed that the expression levels of the cleavage of caspase8, caspase9, caspase3 and PARP were increased in a concentration- and time-dependent manner in choroidal melanoma cells after LiCl treatment (Fig. 3a, b). Annexin V/PI staining was performed to evaluate the effect of LiCl on apoptosis (Fig. 3c, d). Flow cytometry analysis revealed that LiCl induced apoptosis in human choroidal melanoma cells in a concentration-dependent manner. When treated with LiCl (0–40 mM), the frequency of apoptosis increased from 4.30% to 37.60% in OCM1 cells and 8.10% to 39.40% in M619 cells. These findings indicate that treatment with LiCl triggers apoptosis in human choroidal melanoma cells.

### Contribution of the NOXA/Mcl-1 axis to LiCl–induced apoptosis

**Fig. 4:**
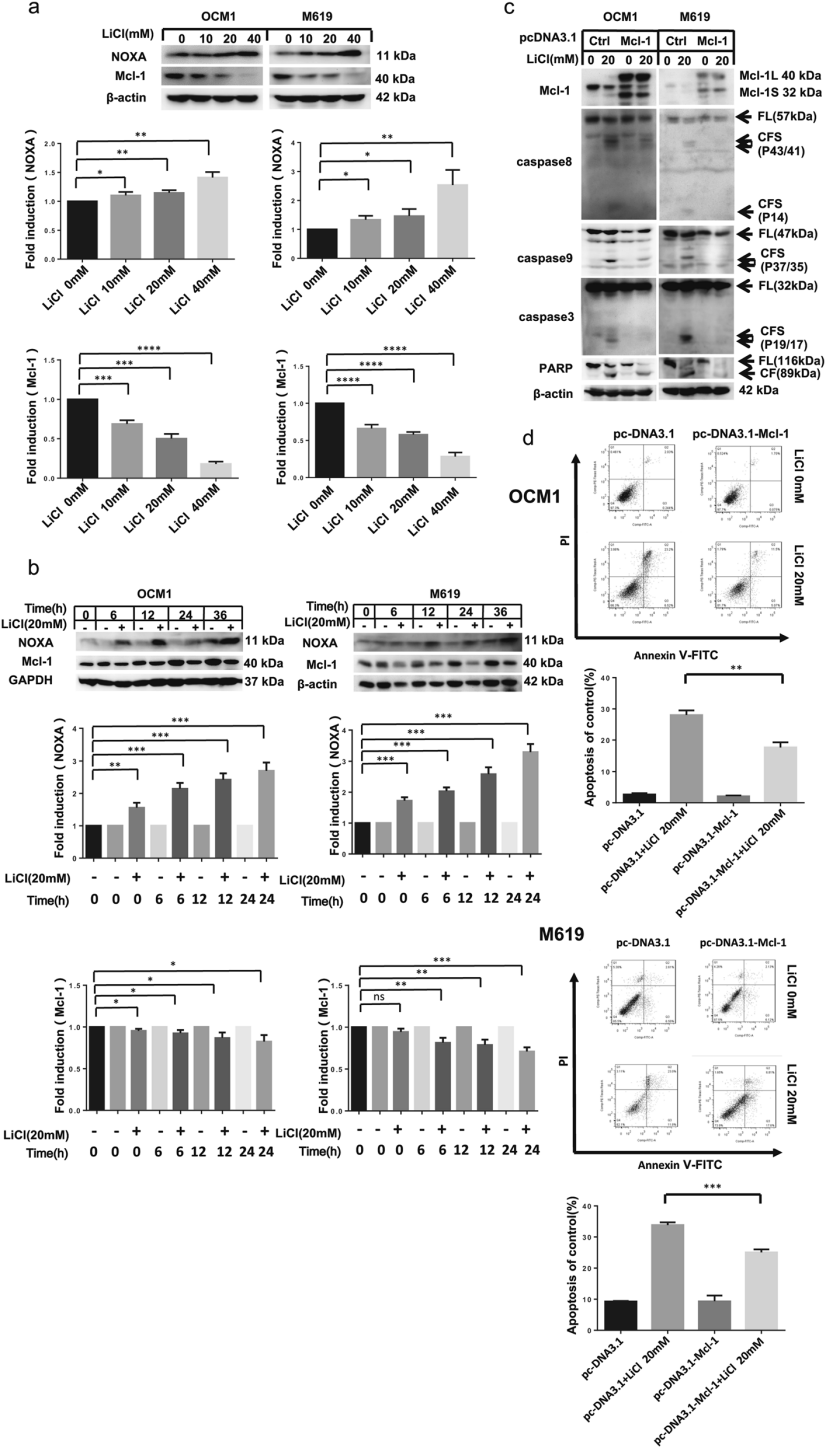
The NOXA/Mcl-1 axis contributes to LiCl-induced apoptosis. **a** OCM1 and M619 cells were treated with 0, 10, 20, or 40 mM LiCl for 36 h and then harvested for western blotting analysis. **b** OCM1 and M619 cells were treated with 20 mM LiCl for 0, 6, 12, 24 and 36 h and then harvested for western blotting analysis. In order to make the comparison more concise and observe the cell, we added a control for 0 h, 6 h, 12 h and 24 h. NOXA and Mcl-1 expression was quantified using ImageJ software and analysed with GraphPad Prism 5.0 software. **c d** OCM1 and M619 cells were seeded in 6-well plates and transfected with vehicle-treated or pc-DNA3.1- Mcl-1 plasmids on the second day. After 48 h of transfection, the cells were exposed to 20 mM LiCl for 24 h and then harvested for western blotting **c** and apoptosis analysis **d**. All data are presented as the mean ± S.D. *P < 0.05, **P < 0.01, ***P < 0.001, ****P < 0.0001

To discover the molecular mechanism of LiCl–induced apoptosis in human choroidal melanoma cells, we analysed the expression of marker proteins in the intrinsic apoptotic signalling pathway after LiCl treatment. The NOXA/Mcl-1 axis has been reported to contribute to chemotherapeutically induced apoptosis In many types of tumour cells [19]. The western blotting results showed that NOXA expression was upregulated in both a concentration- and time-dependent manner following treatment with LiCl, while Mcl-1 expression was downregulated (Fig. 4a, b). In addition, the pcDNA3.1-Mcl-1 plasmid was transfected into OCM1 and M619 cells to confirm whether Mcl-1 downregulation accounted for LiCl–induced apoptosis. As shown in Fig. 4c, Mcl-1 overexpression obviously restrained LiCl-induced cleavage of caspase8, caspase9, caspase3 and PARP. Flow cytometry analysis showed that Mcl-1 overexpression reduced LiCl–induced apoptosis in human choroidal melanoma cells (Fig. 4d). In summary, the data demonstrated that the NOXA/Mcl-1 axis is involved in the anticancer effect of LiCl in choroidal melanoma cells.

### CHOP participated in LiCl–induced activation of NOXA/Mcl-1 axis

**Fig. 5:**
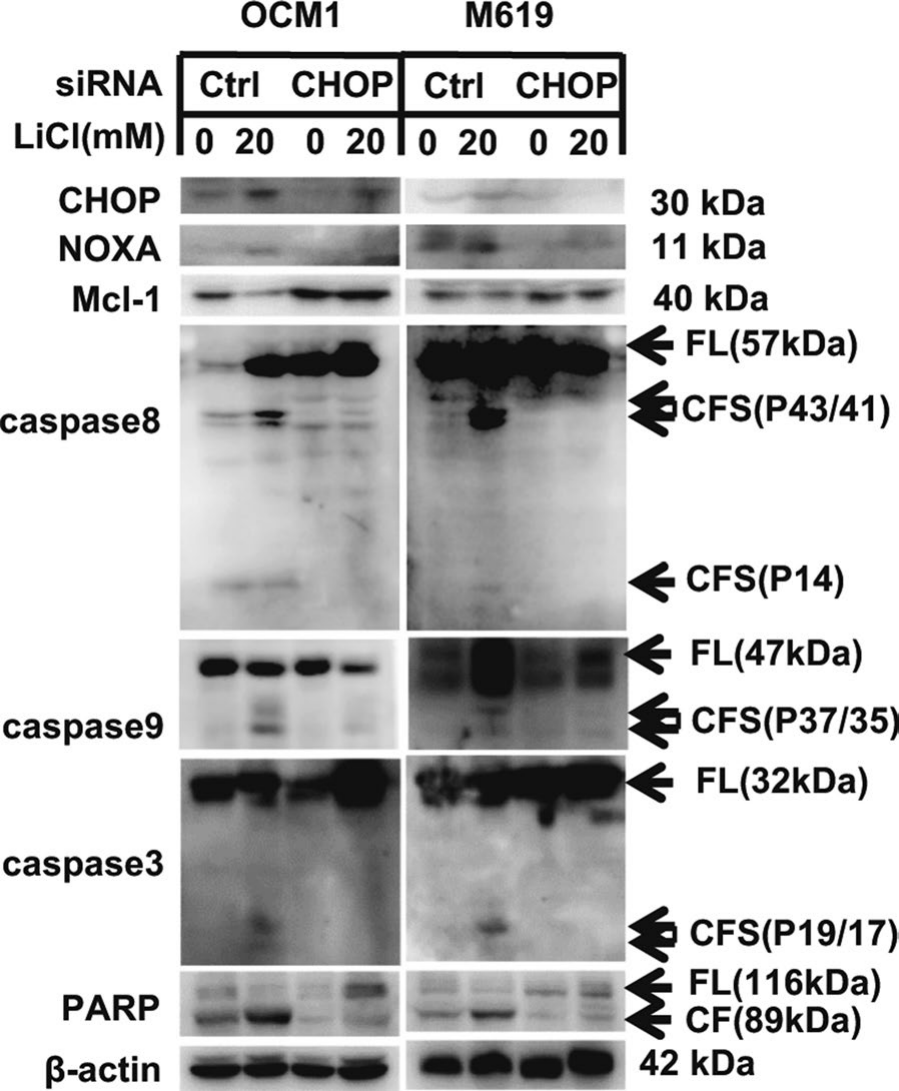
CHOP participates in LiCl-induced apoptosis. OCM1 and M619 cells were seeded in 6-well plates, and on the second day the cells were transfected with control or CHOP siRNA. Two days after transfection, the cells were treated with 0, 20 mM LiCl for another 24 h and then harvested for western blotting analysis

CHOP is an ER stress effector and functions as a bZIP-containing transcription factor that targets several apoptotic genes, including NOXA, regulating their expression and finally resulting in apoptosis. To determine whether ER stress participates in LiCl-induced apoptosis and whether CHOP is involved in LiCl-induced NOXA upregulation, we knocked down CHOP, and detected the expression of NOXA, Mcl-1 and the cleavage of caspases and PARP. Our results showed that when CHOP expression was knocked down and cells were treated with LiCl, the protein level of NOXA was partially increased, and Mcl-1 expression was increased, while the cleavage of caspase8, caspase9, caspase3 and PARP that was induced by the LiCl was reduced compared with the vehicle treated group (Fig. 5). So, CHOP participated in LiCl induced activation of NOXA/Mcl-1 axis. Prolonged ER stress results in the activation of the apoptotic pathway.

### LiCl inhibits choroidal melanoma cell tumorigenesis in vivo

**Fig. 6:**
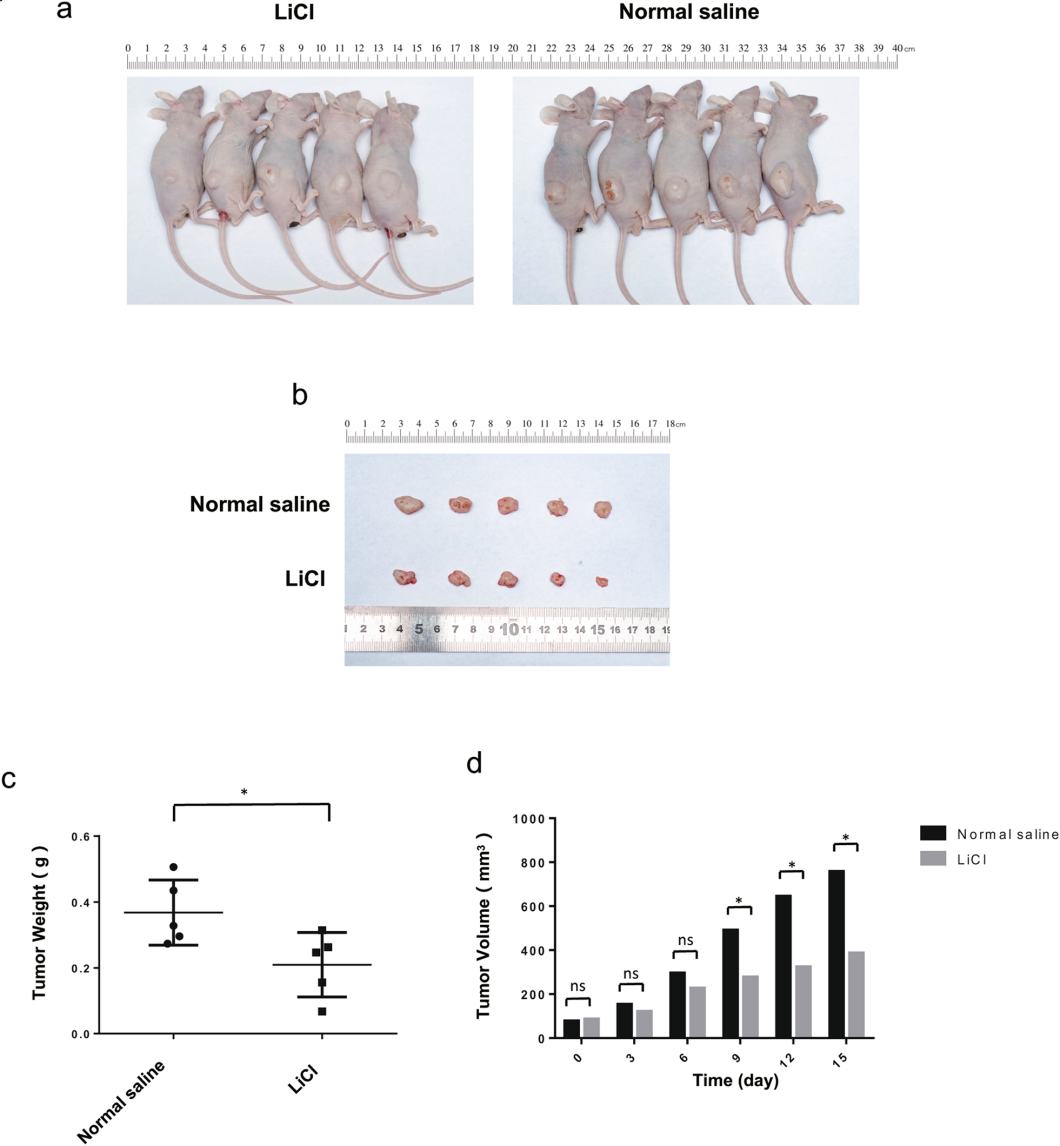
LiCl inhibits choroidal melanoma cell tumorigenesis in vivo. **a** The mouse experiments were performed once. BALB/c nude male mice were randomly divided into a LiCl group and a normal saline group, with 5 mice per group. M619 cells (3 × 10^6^ cells in 100 μl of PBS) were subcutaneously injected into the right flank region of each nude mouse, and then tumours were grown in the mice. The LiCl group was treated with LiCl (141.3 mg/kg; i.p., daily), and the normal saline group received an equal volume of normal saline for 2 weeks. **b, c** At 15 days after inoculation, xenograft tumours were recovered and weighed. **d** The tumour volume was recorded every 3 days beginning on the day at which the tumours were first visible after injection. *P < 0.05. *NS* not significant

**Fig. 7:**
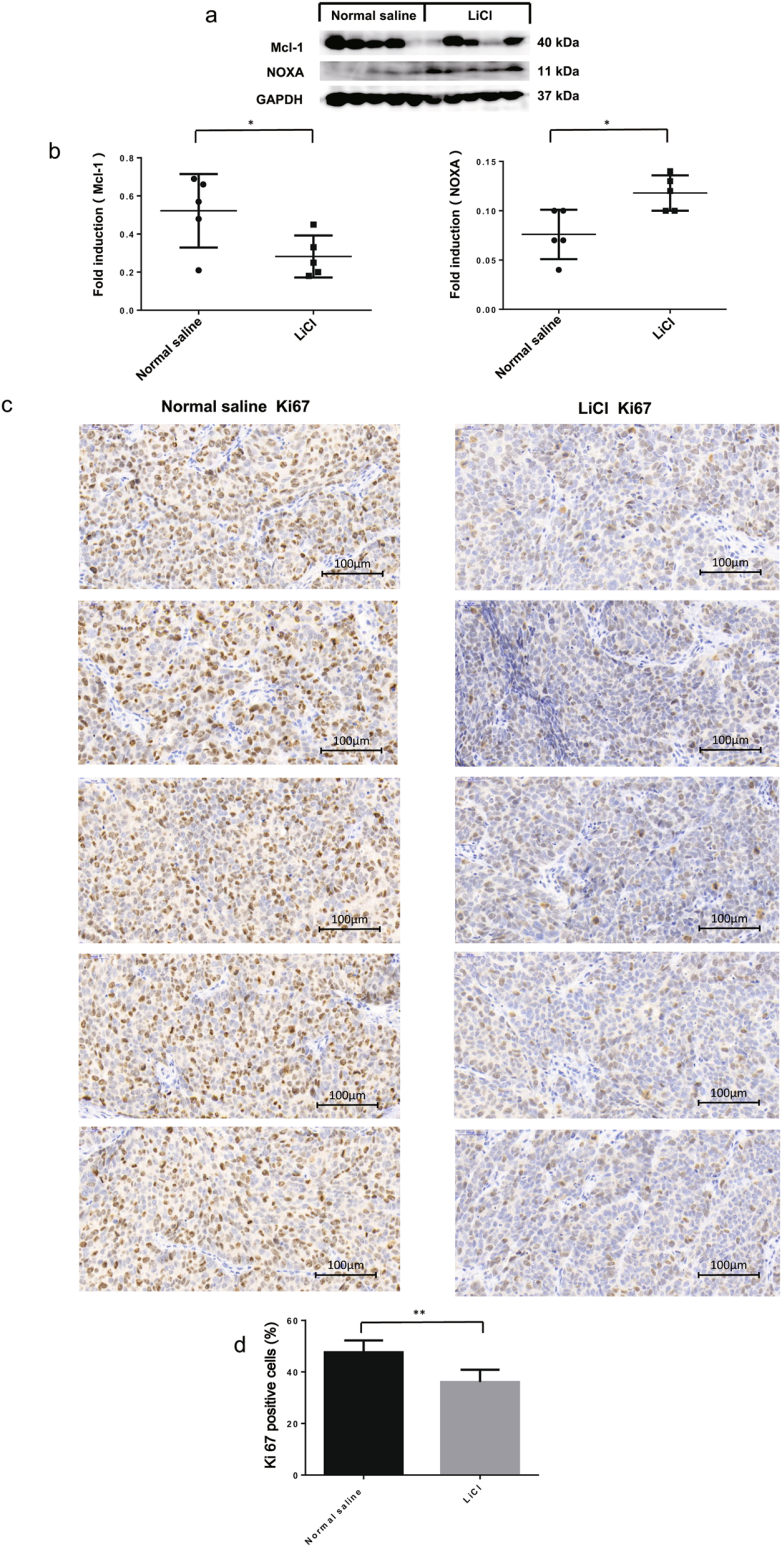
The NOXA/Mcl-1 axis contributes to LiCl-induced apoptosis in vivo. **a b** The nude mice were sacrificed for western blotting and immunohistochemical (IHC) analysis. Western blotting was performed to assess the protein levels of NOXA and Mcl-1 in xenograft tumours. **c** The tumour sections were stained for IHC analysis using antibodies against Ki67 and representative IHC images are shown in figure. Magnification: × 40. Corresponding scale bars are depicted in the lower right corner of each image. Scale bars = 100 μm. **d** The percentages of Ki67-positive cells in the normal saline and LiCl groups. All data are presented as the mean ± S.D. *P < 0.05, **P < 0.01

To determine whether LiCl treatment could affect the tumorigenesis, we transplanted M619 cells into nude mice. The results showed that the tumour size in the LiCl group was significantly smaller than that in the normal saline group (Fig. 6a, b). Moreover, the tumour weight (Fig. 6c) and volume (Fig. 6d) were noticeably decreased in the LiCl group compared with the normal saline group at 15 days after inoculation. Western blotting analysis revealed that tumour tissues from the LiCl group displayed higher levels of NOXA and lower levels of Mcl-1 than those from the normal saline group (Fig. 7a, b). These results indicate that LiCl inhibits choroidal melanoma cell tumorigenesis in vivo and that the NOXA/Mcl-1 axis contributes to this inhibitory effect. To further investigate the mechanism by which LiCl inhibits tumour growth, the expression levels of a proliferative marker (Ki67) were determined by immunohistochemical analysis. The results showed a decrease in Ki67 expression in tumours from mice treated with LiCl compared to normal saline mice (Fig. 7c). Overall, these results were consistent with the in vitro results and showed that LiCl was an efficacious antitumour drug in the xenograft model. The mouse weights and tumour volumes are shown in Table 1.

**Table 1:** The body weights (**a**) and the tumour volumes (**b**) of the nude mice

Group Date	1/10	1/13	1/16	1/19	1/22	1/25
a Mice weight(g)						
Normal saline						
1	14.57	15.46	16.68	17.18	16.88	18.00
2	17.75	17.10	19.80	20.00	20.01	21.00
3	15.62	15.48	16.91	17.37	17.39	18.23
4	14.42	14.16	16.47	16.94	16.39	18.78
5	13.68	15.40	16.03	16.53	15.53	17.63
LiCl						
1	16.86	17.43	19.08	18.75	17.44	19.28
2	16.90	16.23	17.85	18.33	17.90	19.47
3	13.11	15.26	16.66	17.36	17.03	18.14
4	16.88	17.61	19.19	19.35	17.89	18.05
5	13.53	15.30	16.90	17.11	16.32	17.24
B Tumor volume (mm3)						
Normal saline						
1						
L	6.86	8.04	9.11	11.03	12.26	13.14
W	4.73	5.62	7.70	9.47	9.82	10.15
V	76.74	126.50	270.07	494.59	591.13	676.86
2						
L	6.49	7.86	8.70	10.02	11.97	12.19
W	4.62	5.67	7.61	10.84	10.43	11.54
V	69.26	126.35	251.92	544.17	651.08	811.68
3						
L	6.00	8.27	9.95	11.59	13.06	13.60
W	5.97	6.33	8.14	8.77	9.39	9.91
V	106.92	165.68	329.64	445.71	575.76	667.82
4						
L	6.11	9.88	11.35	13.82	14.89	16.90
W	4.59	7.30	8.91	9.55	10.86	10.91
V	64.36	263.25	450.53	630.21	878.06	1005.79
5						
L	6.48	5.40	8.00	10.35	11.45	12.45
W	4.53	5.58	6.62	8.05	9.58	10.02
V	66.49	81.36	175.30	335.35	525.42	624.99
LiCl						
1						
L	6.48	7.98	9.74	9.82	11.01	11.22
W	5.61	6.63	8.28	9.15	9.35	9.99
V	101.97	175.39	333.88	411.08	481.26	559.87
2						
L	5.45	5.60	5.93	5.58	6.52	6.62
W	5.45	5.40	5.36	5.57	5.13	5.09
V	80.94	81.65	85.18	86.56	85.79	85.76
3						
L	6.14	7.23	8.04	10.34	11.59	12.20
W	5.21	5.23	7.76	8.27	8.53	8.90
V	83.33	98.88	242.07	353.59	421.65	483.18
4						
L	5.09	5.87	6.13	7.21	7.33	8.05
W	5.09	5.62	6.12	6.45	8.00	8.21
V	65.94	92.70	114.80	149.98	214.92	266.01
5						
L	5.82	6.58	8.72	8.94	9.18	10.14
W	5.82	7.07	9.35	9.58	9.83	10.44
V	98.57	153.05	355.48	382.83	414.19	536.71

## Discussion

Choroidal melanoma is a serious metastatic malignant melanoma with poor prognosis. Common treatments for choroidal melanoma offer only temporary relief and are ineffective in inhibiting tumour metastasis or improving the survival rate [20]. Thus, exploring new treatment strategies is important for improving the prognosis of choroidal melanoma patients [8].

LiCl has been safely used in the clinic for the treatment of psychiatric disorders for several decades [21]. LiCl has also been reported to exhibit antitumour effects in various cancers [12]. As a GSK3β inhibitor, the most robust antineoplastic effect of lithium is related to GSK3β [22]. However, other study showed that LiCl exerts antitumour effects that are independent of GSK3β [13]. The present study investigated whether LiCl exhibited antitumour effects in choroidal melanoma cells and demonstrated the underlying molecular mechanism.

In this study, we evaluated the effect of LiCl on choroidal melanoma cell survival and colony formation and provided a potential mechanism. We analysed the cytotoxicity of LiCl against choroidal melanoma cells and found that LiCl displayed a concentration- and time-dependent inhibitory effect on the survival of choroidal melanoma cells. Furthermore, the number of colonies formed notably declined with increasing concentrations of LiCl.

As shown in Additional file 1: Figure S1, the OCM1 and M619 cells were treated with different doses of NaCl or KCl at the same concentration of LiCl for different times. Similar survival inhibition effect of NaCl and KCl on the human choroidal melanoma cells were not detected. The normal 293T cells were also treated with 0, 2.5, 5, 10, 20, 40 mM LiCl for 24 h,36 h and 48 h respectively. LiCl exerts no inhibition effect on normal cells even at the highest concentration of 40 mM. In conclusion, LiCl could be considered as a potential anticancer agent.

It has been demonstrated that the endoplasmic reticulum (ER) response is activated when cancer cells treated with chemotherapeutics [18]. We examined relevant proteins in the ER stress pathway to confirm whether LiCl triggers ER stress in choroidal melanoma cells. The western blotting results demonstrated that expression of the marker proteins IRE1α, Bip, p-eIF2α, ATF4 and CHOP were upregulated in a concentration-dependent manner after LiCl treatment. These data indicate that LiCl triggers ER stress in choroidal melanoma cells.

Flow cytometry analysis revealed that LiCl induced apoptosis in choroidal melanoma cells in a concentration-dependent manner. Subsequently, it was demonstrated that the levels of the cleaved forms of apoptosis-associated proteins were distinctly increased both in a dose- and time-dependent manner following LiCl treatment of OCM1 and M619 cells. These results revealed that LiCl exerts anticancer effects by decreasing cell viability, inhibiting colony formation, and inducing caspase-dependent apoptosis in choroidal melanoma cells. (Additional file 1: Figure S2).

Apoptosis, known as programmed cell death, suppressescarcinogenesis in normal cells by regulating expression of cancer-related genes [23]. As an anti-apoptotic protein, Mcl-1 belongs to the BCL-2 family, which is closely connected to inhibitions of mitochondrial apoptosis [24]. Blocking Mcl-1 makes tumour cells more susceptible to anticancer agents [25]. NOXA, a BH3-only protein in the Bcl-2 family, has been reported to participate in chemotherapy-induced apoptosis in melanoma [26]. Interactions between Mcl-1 and NOXA regulate the mitochondrial apoptotic pathway [27]. In the present study, we found that after LiCl treatment, the level of Mcl-1 was obviously decreased, while the protein NOXA was upregulated in a concentration- and time-dependent manner. We found that Mcl-1 overexpression dramatically weakened LiCl-induced cleavage of apoptosis-associated proteins and impaired apoptosis after drug treatment. These results demonstrated that the NOXA/Mcl-1 axis contribute to LiCl-induced intrinsic mitochondrial apoptosis in choroidal melanoma cells.

A previous study showed that cell apoptosis may be induced by ER stress and mitochondrial membrane permeability [28, 29]. CHOP, a key protein of ER stress-mediated cell death, also known as a bZIP-containing transcription factor, targets many apoptotic genes, including NOXA, modifying their expression and ultimately resulting in apoptosis [30]. Western blot showed that when CHOP was knocked down, the NOXA expression was decreased, and the cleavage of caspases and PARP were simultaneously weakened compared with control siRNA-transfected cells after the treatment of LiCl. These data indicate that the ER stress participates in the LiCl-induced apoptosis and CHOP was involved in LiCl-induced NOXA upregulation in choroidal melanoma cells.

GSK3β plays multiple roles in different cancers, but its importance is still controversial [31]. Inactivation of GSK3β by LiCl sensitizes both hepatoma and prostate cancer to TRAIL-induced apoptosis [32]. However, other study indicated that LiCl significantly enhanced cell apoptosis in non-small cell lung cancer by upregulating the death receptors DR4 and DR5, and LiCl sensitized cells to TRAIL-induced apoptosis independent of GSK3β [13]. We have knocked down the GSK3β through transfecting siRNA, the result showed that there was no change in the expression of NOXA, Mcl-1 and the cleavage of caspases and PARP. Therefore, we considered that LiCl-induced apoptosis in human choroidal melanoma cells was GSK3β independent. However, whether DR4 and DR5 involved in LiCl induced apoptosis in human choroidal melanoma cells or not requires further research.

To further investigate whether LiCl could affect tumorigenesis, we transplanted M619 cells into nude mice and found that xenograft tumour growth in the LiCl group was significantly slower than that in the normal saline group. At 15 days after inoculation, tumour size and weight were dramatically decreased in the LiCl group. Ki-67, a nuclear protein, is widely used as a tumour proliferation marker. Our study showed that the number of Ki67-positive cells from the xenograft tumours also declined in the LiCl group. Furthermore, LiCl treatment significantly reduced Mcl-1 expression and upregulated the NOXA level in M619 cell-based xenografts. In summary, LiCl prevents xenograft tumour growth in mice, and the NOXA/Mcl-1 axis is associated with this effect.

## Conclusion

In summary, we demonstrate that LiCl exerts apoptotic effects on choroidal melanoma cells. The NOXA/Mcl-1 axis is involved in LiCl-induced intrinsic apoptosis both in vitro and in vivo. Furthermore, LiCl triggers ER stress in choroidal melanoma cells to induce intrinsic apoptosis. LiCl inhibits choroidal melanoma cell tumorigenesis in vivo. This study may provide an important theoretical basis for promoting LiCl as a potential clinical therapeutic strategy to treat choroidal melanoma.

## Supplementary information

**Additional file 1: Fig. S1.** NaCl and KCl didn’t exerts obvious survival inhibition effect on the human choroidal melanoma cells and LiCl exerts no inhibition effect on normal cells. **a** OCM1 and **b** M619 cells were seeded in 96-well plates, treated with 0, 2.5, 5, 10, 20 or 40 mM NaCl and incubated for 24 h, 36 h, 48 h. **c** OCM1 and **d** M619 cells were seeded in 96-well plates, treated with 0, 2.5, 5, 10, 20 or 40 mM NaCl and incubated for 24 h, 36 h, 48 h. **e** 293T cells were seeded in 96-well plates, treated with 0, 2.5, 5, 10, 20 or 40 mM LiCl and incubated for 24 h, 36 h, 48 h. Cell survival was examined using the MTT assay. The survival rate at each drug concentration was compared with that of the normal saline group and analysed using SPSS software. All data are presented as the mean ± S.D. ns: not significant

**Additional file 2: Fig. S2.** LiCl-induced apoptosis in human choroidal melanoma cells was GSK3β independent. OCM1 and M619 cells were seeded in 6-well plates, and on the second day the cells were transfected with control or GSK3β siRNA. Two days after transfection, the cells were treated with 0, 20 mM LiCl for another 24 h and then harvested for western blotting analysis

## Data Availability

All data generated or analysed during this study are included in this published article.

## Abbreviations

LiCl: Lithium chloride
PARP: Poly(ADP-ribose) polymerase
PBS: Phosphate-buffered saline
PI: Propidium iodide
ECL: Enhanced chemiluminescence
IHC: Immunohistochemical
DAB: Diaminobenzidine
ER: Endoplasmic reticulum
L: Length
W: Width
V: Volume
NS: Not significant
CF: Cleaved form
CFs: Plural of cleaved form

## References

[1] Siegel RL, Miller KD, Jemal A (2019). Cancer statistics, 2019. CA Cancer J Clin.

[2] Dogrusoz M, Jager MJ, Damato B (2017). Uveal Melanoma Treatment and Prognostication. Asia Pac J Ophthalmol (Phila).

[3] Hamal D (2019). Choroidal Melanoma: our Experience. Nepal J Ophthalmol.

[4] Lorenzo D (2019). Clinical predictors of survival in metastatic uveal melanoma. Jpn J Ophthalmol.

[5] Mahendraraj K (2016). Trends in incidence, survival, and management of uveal melanoma: a population-based study of 7,516 patients from the Surveillance, Epidemiology, and End Results database (1973-2012). Clin Ophthalmol.

[6] Papakostas TD (2017). Long-term outcomes after proton beam irradiation in patients with large choroidal melanomas. JAMA Ophthalmol.

[7] Hamza HS, Elhusseiny AM (2018). Choroidal Melanoma Resection. Middle East Afr J Ophthalmol.

[8] Asadi S (2015). Gold nanoparticle-based brachytherapy enhancement in choroidal melanoma using a full Monte Carlo model of the human eye. J Appl Clin Med Phys.

[9] Shorter E (2009). The history of lithium therapy. Bipolar Disord.

[10] Cohen P, Goedert M (2004). GSK3 inhibitors: development and therapeutic potential. Nat Rev Drug Discov.

[11] Eiraku N (2019). BMP9 directly induces rapid GSK3-beta phosphorylation in a Wnt-independent manner through class I PI3K-Akt axis in osteoblasts. FASEB J.

[12] Li L (2015). Lithium Chloride Promotes Apoptosis in Human Leukemia NB4 Cells by Inhibiting Glycogen Synthase Kinase-3 Beta. Int J Med Sci.

[13] Lan Y (2013). Lithium enhances TRAIL-induced apoptosis in human lung carcinoma A549 cells. Biometals.

[14] Gajos-Michniewicz, A. and M. Czyz, *WNT Signaling in Melanoma.* Int J Mol Sci, 2020. **21**(14). 10.3390/ijms21144852 PMC7402324 32659938

[15] Zhao X, Liu X, Su L (2014). Parthenolide induces apoptosis via TNFRSF10B and PMAIP1 pathways in human lung cancer cells. J Exp Clin Cancer Res.

[16] Park YL (2019). Activation of WNT/beta-catenin signaling results in resistance to a dual PI3K/mTOR inhibitor in colorectal cancer cells harboring PIK3CA mutations. Int J Cancer.

[17] Cheng S (2018). HOXA4, down-regulated in lung cancer, inhibits the growth, motility and invasion of lung cancer cells. Cell Death Dis.

[18] Zhang Q (2019). Piperlongumine, a Novel TrxR1 inhibitor, induces apoptosis in hepatocellular carcinoma cells by ROS-mediated ER stress. Front Pharmacol.

[19] Zhao X (2017). c-FLIP and the NOXA/Mcl-1 axis participate in the synergistic effect of pemetrexed plus cisplatin in human choroidal melanoma cells. PLoS ONE.

[20] Wang Y (2013). Efficacy and safety of dendrimer nanoparticles with coexpression of tumor necrosis factor-alpha and herpes simplex virus thymidine kinase in gene radiotherapy of the human uveal melanoma OCM-1 cell line. Int J Nanomedicine.

[21] Cohen P, Goedert M (2004). GSK3 inhibitors: development and therapeutic potential. Nature Rev Drug Discovery.

[22] Gupta A (2011). Interaction networks of lithium and valproate molecular targets reveal a striking enrichment of apoptosis functional clusters and neurotrophin signaling. Pharmacogenomics J.

[23] Qian B (2020). Hic-5 in pancreatic stellate cells affects proliferation, apoptosis, migration, invasion of pancreatic cancer cells and postoperative survival time of pancreatic cancer. Biomed Pharmacotherapy.

[24] Senichkin VV (2019). Molecular comprehension of Mcl-1: from gene structure to cancer therapy. Trends Cell Biology.

[25] Akgul C (2008). Mcl-1 is a potential therapeutic target in multiple types of cancer. Cellular Molecular Life Sci.

[26] Weber, A., et al., Endogenous Noxa Determines the Strong Proapoptotic Synergism of the BH3-Mimetic ABT-737 with Chemotherapeutic Agents in Human Melanoma Cells. Translational Oncology, 2009. **2**(2): p. 73-IN5. 10.1593/tlo.08223 PMC2670574 19412422

[27] Mazumder S (2012). Mcl-1 phosphorylation defines ABT-737 resistance that can be overcome by increased NOXA expression in leukemic B cells. Cancer Res.

[28] Wang, H., et al., Apoptosis and necrosis induced by novel realgar quantum dots in human endometrial cancer cells via endoplasmic reticulum stress signaling pathway. International Journal of Nanomedicine, 2015: p. 5505. 10.2147/IJN.S83838 PMC4560518 26357474

[29] Arakawa S (2015). Identification of a novel compound that inhibits both mitochondria-mediated necrosis and apoptosis. Biochem Biophysical Res Commun.

[30] Jin HR (2014). Anticancer compound Oplopantriol A kills cancer cells through inducing ER stress and BH3 proteins Bim and Noxa. Cell Death Dis.

[31] McCubrey JA (2014). GSK-3 as potential target for therapeutic intervention in cancer. Oncotarget.

[32] Domoto, T., et al., Glycogen Synthase Kinase 3beta in Cancer Biology and Treatment. Cells, 2020. **9**(6). 10.3390/cells9061388 PMC7349761 32503133

