# Supplementary figures and images for: LiCl induces apoptosis via CHOP/NOXA/Mcl-1 axis in human choroidal melanoma cells
Source: Cancer Cell Int. 2021 Feb 8;21:96. doi: 10.1186/s12935-021-01778-2 (PMC7869481; doi:10.1186/s12935-021-01778-2)

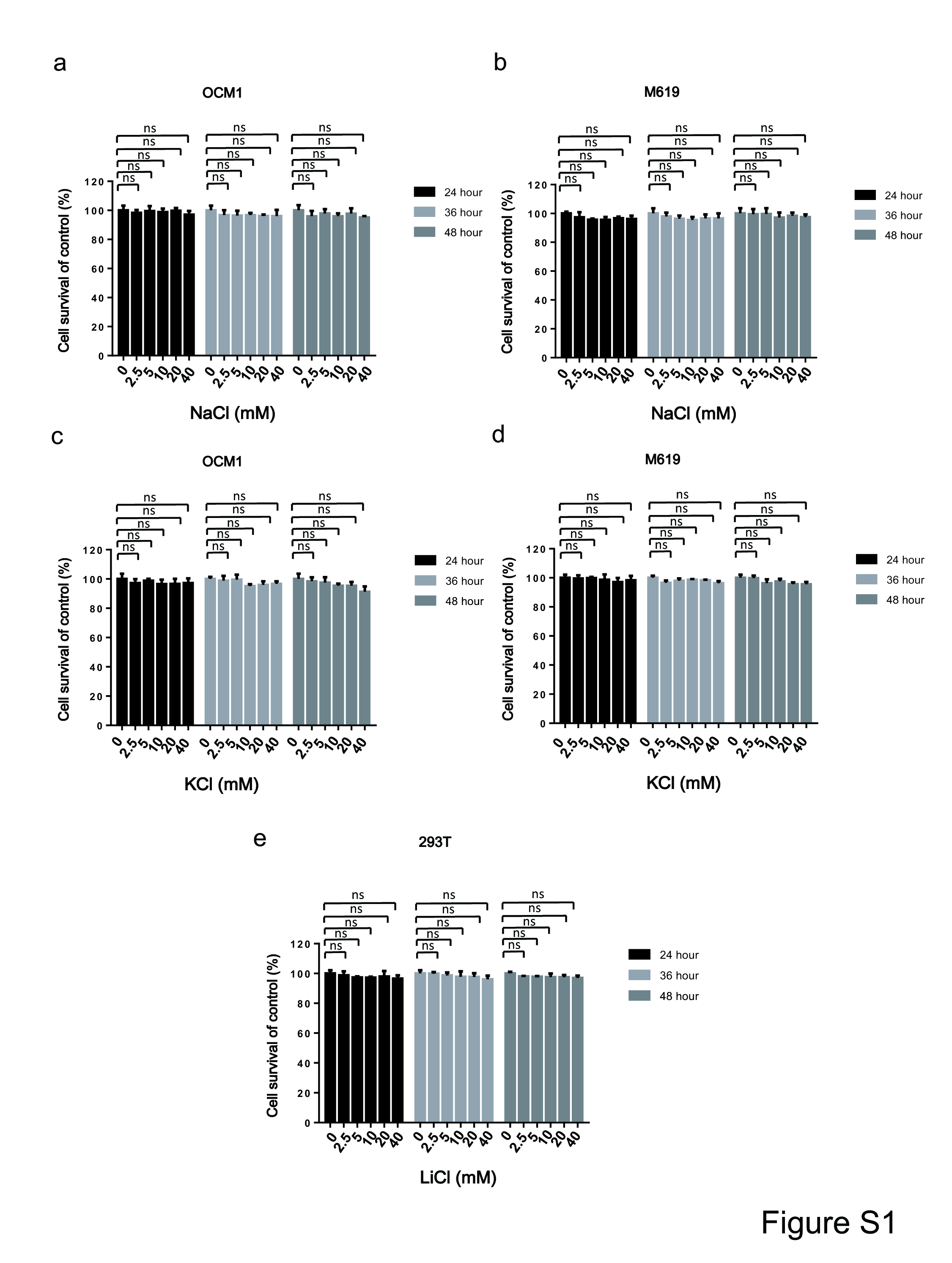

Supplement: Supplementary file 1 — Additional file 1: Fig. S1. NaCl and KCl didn’t exerts obvious survival inhibition effect on the human choroidal melanoma cells and LiCl exerts no inhibition effect on normal cells. a OCM1 and b M619 cells were seeded in 96-well plates, treated with 0, 2.5, 5, 10, 20 or 40 mM NaCl and incubated for 24 h, 36 h, 48 h. c OCM1 and d M619 cells were seeded in 96-well plates, treated with 0, 2.5, 5, 10, 20 or 40 mM NaCl and incubated for 24 h, 36 h, 48 h. e 293T cells were seeded in 96-well plates, treated with 0, 2.5, 5, 10, 20 or 40 mM LiCl and incubated for 24 h, 36 h, 48 h. Cell survival was examined using the MTT assay. The survival rate at each drug concentration was compared with that of the normal saline group and analysed using SPSS software. All data are presented as the mean ± S.D. ns: not significant [file 12935_2021_1778_MOESM1_ESM.tif]

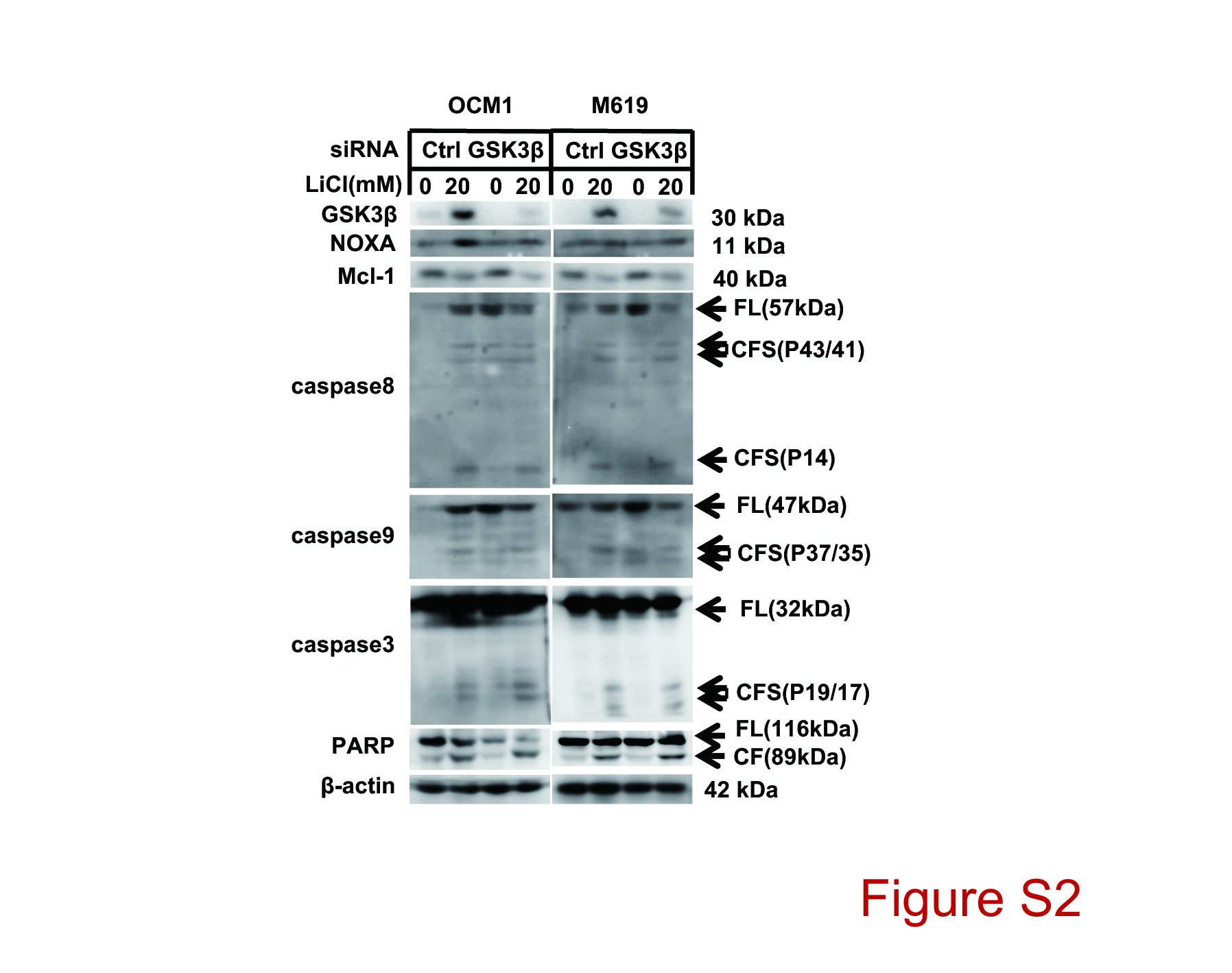

Supplement: Supplementary file 2 — Additional file 2: Fig. S2. LiCl-induced apoptosis in human choroidal melanoma cells was GSK3β independent. OCM1 and M619 cells were seeded in 6-well plates, and on the second day the cells were transfected with control or GSK3β siRNA. Two days after transfection, the cells were treated with 0, 20 mM LiCl for another 24 h and then harvested for western blotting analysis [file 12935_2021_1778_MOESM2_ESM.tif]
